# Simultaneous arthroscopic cystectomy and unicompartmental knee arthroplasty for the management of partial knee osteoarthritis with a popliteal cyst: A case report

**DOI:** 10.3389/fsurg.2023.1109571

**Published:** 2023-03-30

**Authors:** Cai Liu, Dejie Zhou, Xinwei Liu, Jin Huang, Jianguo Fang, Hongyu Zhou, Jianjun Luo, Yiqian Luo, Lianghu Zhao

**Affiliations:** ^1^ Department of Orthopedic Surgery, The Affiliated Hospital of Panzhihua University, Panzhihua, China; ^2^ Department of Physical Examination Center, The Affiliated Hospital of Panzhihua University, Panzhihua, China; ^3^ Department of Spine Surgery, Yangpu District Shidong Hospital Affiliated to the University of Shanghai for Science and Technology, Shanghai, China

**Keywords:** Baker's cyst, popliteal cyst, unicompartmental knee arthroplasty, arthroscopic cystectomy, unicompartmental arthroplasty

## Abstract

**Introduction:**

Popliteal cysts are secondary to degenerative changes in the knee joint. After total knee arthroplasty (TKA), 56.7% of patients with popliteal cysts at 4.9 years follow-up remained symptomatic in the popliteal area. However, the result of simultaneous arthroscopic cystectomy and unicompartmental knee arthroplasty (UKA) was uncertain.

**Case presentation:**

A 57-year-old man was admitted to our hospital with severe pain and swelling in his left knee and the popliteal area. He was diagnosed with severe medial unicompartmental knee osteoarthritis (KOA) with a symptomatic popliteal cyst. Subsequently, arthroscopic cystectomy and unicompartmental knee arthroplasty (UKA) were performed simultaneously. A month after the operation, he returned to his normal life. There was no progression in the lateral compartment of the left knee and no recurrence of the popliteal cyst at the 1-year follow-up.

**Conclusion:**

For KOA patients with a popliteal cyst seeking UKA, simultaneous arthroscopic cystectomy and UKA are feasible with great success if managed appropriately.

## Introduction

Popliteal cysts were first discovered by Adama in 1840 and first described in detail by Baker in 1877 and are now commonly known as Baker's cysts (1, 2). Popliteal cysts are the most prevalent cystic lesions around the knee, originating from the gastrocnemius-semimembranosus bursa, with the unidirectional communications between the cysts and the knee joints (3, 4).

Popliteal cysts are a secondary phenomenon associated with the overproduction of synovial fluid, especially in patients with knee osteoarthritis (KOA), intra-articular inflammation, or cartilage/meniscal pathology, prevalence ranging from 4.7% to 58.0% (5–8). Historically, patients with KOA and popliteal cysts were treated with isolated knee arthroplasty (TKA) procedures (7, 9). However, potential cysts were still detectable in 85.3% of these patients at 1-year follow-up, and 33% at mid-term follow-up (4.9 years). Furthermore, 56.7% of these patients were still symptomatic at mid-term follow-up (9). Therefore, we aimed to evaluate the effectiveness of simultaneous cystectomy and unicompartmental knee arthroplasty (UKA). To our knowledge, this is the first case report of a KOA patient with a popliteal cyst who underwent arthroscopic popliteal cystectomy and UKA simultaneously.

## Case presentation

A 57-year-old non-smoking man with a BMI of 30 was admitted to our hospital with severe pain (VAS: 7) and swelling in his left knee and the popliteal area. He was diagnosed with KOA 5 years prior. He had tried the regular conservative treatments by taking glucosamine hydrochloride and diacerein for 3 months, and loxoprofen sodium tablets for 1 week. Before coming to our department, he had tried various integrated traditional Chinese and Western medicine treatments, including acupuncture, moxibustion, and a small needle knife. He also had the aspiration of the popliteal cyst with the injection of the compound betamethasone once and Sodium Hyaluronate in the knee joint for 3 cycles (1 cycle: 1 injection once a week for 5 weeks). During this period, he also insisted on doing quadriceps functional exercises. However, pain relief in the knee and the popliteal area was minimal, and the remission time was gradually shortened. He had no other medical morbidities. Physical examination revealed five old healed skin burns caused by moxibustion around the knee, varus malalignment of the left knee with reduced flexion (30° compared with the contralateral), swelling in the suprapatellar and popliteal area, and positive of the Ballotman's and McMurray's tests, no instability was found. The pain was focused along the medial joint line and posterior medial knee exacerbated with deep knee flexion without evidence of patellofemoral or lateral joint line pain.

**Figure 1:**
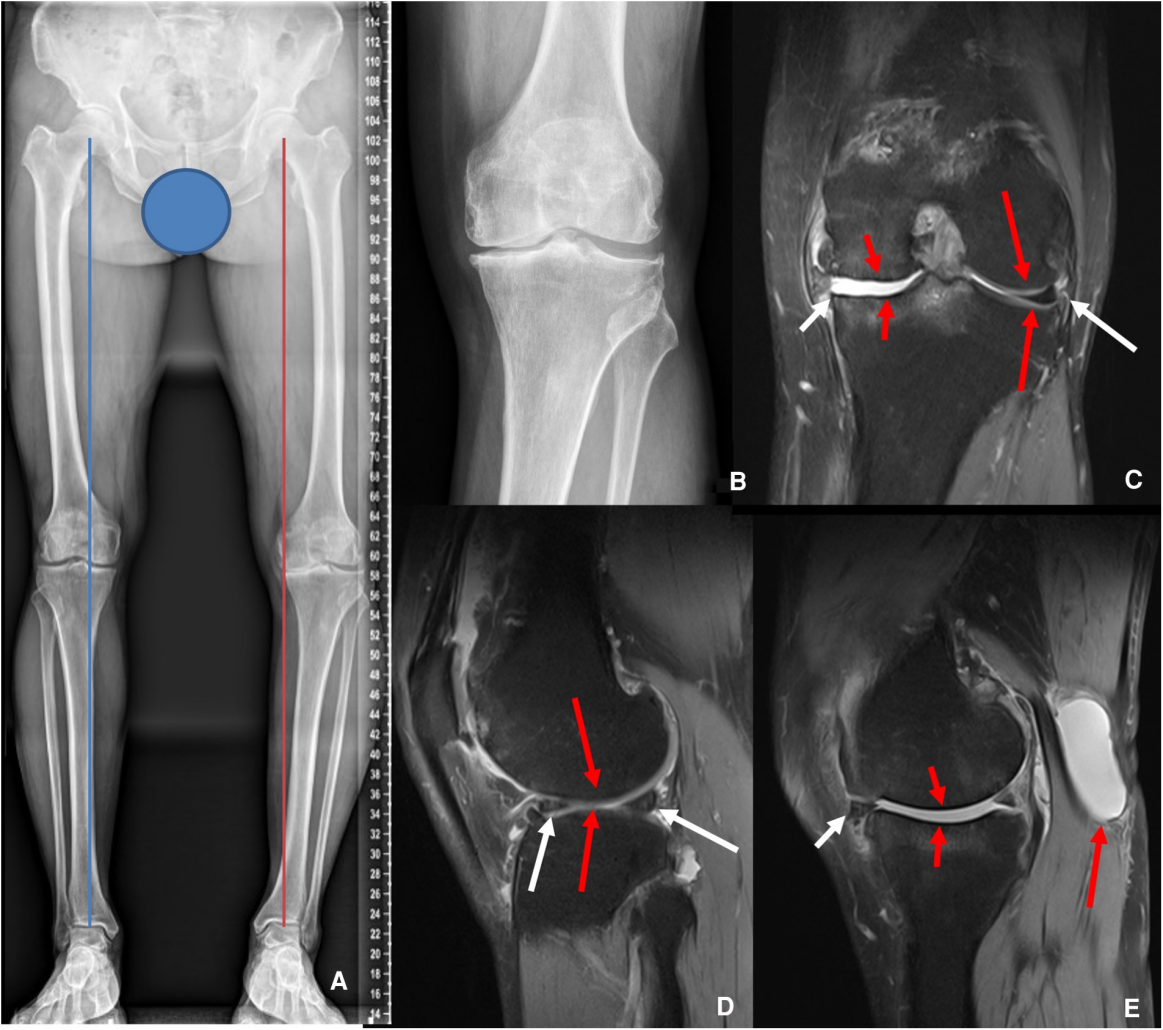
Preoperative X-ray and MRI. (**A**) The left Weight-Bearing line (red) was 35 mm more varus than the contralateral side (blue). (**B**) Narrowing of the medial compartment of the left knee. (**C,D**) The short white arrow showed subluxation of the medial meniscus. The short red arrows showed the cartilage wearing on both sides of the medial femur and tibia. The long red arrows showed the normal cartilage on the lateral side of the knee. The long white arrows showed the lateral meniscus was intact. (**E**) The short white arrow showed subluxation of the medial meniscus. The short red arrows showed the cartilage wearing. The long red arrow indicated the popliteal cyst.

Preoperative ultrasonography showed suprapatellar bursa effusion and a large fluid bursa (Length: 5.08 cm, depth: 1.76 cm, width: 2.60 cm) between the semimembranosus and gastrocnemius tendons, and with no evidence of deep vein thrombosis. The anteroposterior weight-bearing plain radiographs revealed a significant varus malalignment with the deviation of the mechanical axis (MAD) 35 mm more than normal in the left lower extremity, and there were no abnormal angles in the anatomical lateral distal femur and anatomical medial proximal tibia (10, 11). Kellgren-Lawrence (K/L) medial compartment grade IV and lateral compartment K/L grade II were based on radiographs of the left knee (Figures 1A,B) (12). Preoperative T2-weighted magnetic resonance imaging (MRI) showed subluxation of the medial meniscus. Server cartilage wearing on both sides of the medial femur and tibia of the knee joint was found. There was a high-signal-intensity cyst (Length: 5.27 cm, depth: 1.79 cm, width: 3.10 cm) between the semimembranosus and the medial head of the gastrocnemius tendons (Figures 1C–E). The intact anterior cruciate ligament (ACL) was shown normally on MRI. The collateral ligaments were intact. The laboratory data were all in normal ranges.

**Figure 2:**
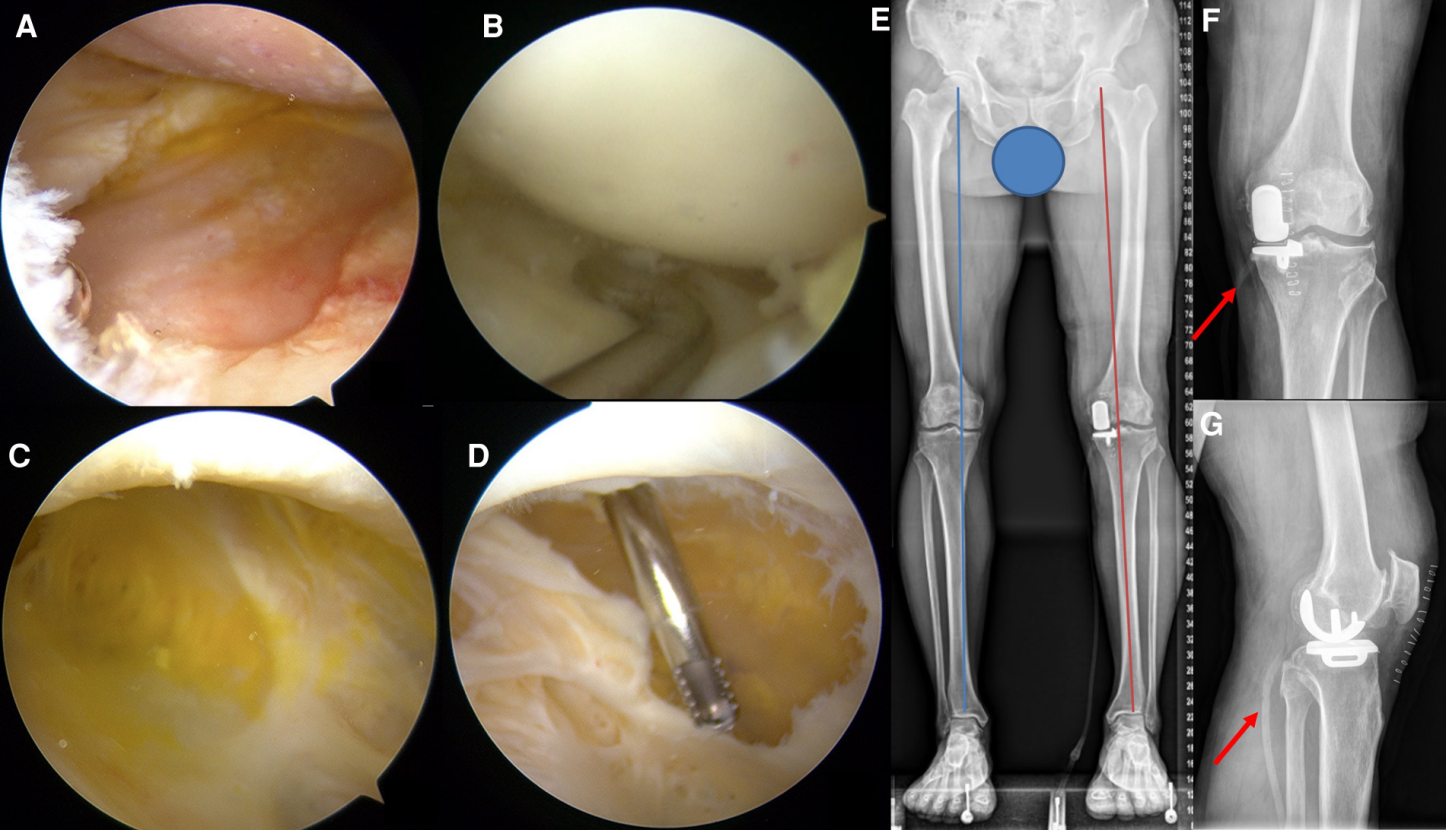
Arthroscopic surgery view (**A–D**) and immediate postoperative X-ray (**E–G**) evaluation after the surgery. (**A**) Severe cartilage wearing of the medial femoral and tibial condyles. (**B**) Normal lateral meniscus and cartilage of the femoral and tibial condyles. (**C**) The wall of the popliteal cyst is filled with orange juice-like fluid. (**D**) Resection of the cyst wall. (**E**) The Weight-Bearing line (red) of the left lower extremity returned to a normal position similar to the contralateral side (blue). (**F,G**) Long red arrows indicated the drainage tube.

The senior surgeon (Jin Huang) performed a diagnostic arthroscopy with a 30-degree arthroscope *via* the anteromedial and anterolateral portals, with a tourniquet in the supine position. Obvious synovial hyperplasia was visualized under arthroscopy and numerous osteochondral defects were found both in the medial femoral and tibial condyles, with server medial meniscal damage (Figure 2A). However, the cartilage on the lateral femoral and tibial condyle and the lateral meniscus remained intact (Figure 2B). The posteromedial corner of the joint was then exposed via the posteromedial portal entrance to identify the capsular fold (13). The uni-valvular connection between the semimembranosus and medial head of the gastrocnemius tendons to the cyst was exposed after enlarging the capsular fold. The intact cyst wall was visualized by switching the arthroscope to the posteromedial portal and filled with orange juice-like fluid (Figure 2C). The fluid aspired for smear and bacterial culture examination through the operating portal, and the results were negative. The following completely dissected cyst wall was performed *via* an additional portal inferior and posterior to the posteromedial portal, and a drainage tube was used (Figures 2D–G).

Then, the UKA was performed in the previous supine position with additional disinfection with iodophor in the surgery region. The knee joint was exposed through a medial parapatellar approach. Extensive osteochondral defects in the medial femur and tibia condyle were confirmed, with medial meniscal damage. In contrast, the lateral cartilaginous bone, meniscus, and ACL were intact. The rest of the procedures were performed according to the medial UKA operation manual. 2 g of cefazolin was used 30 min before and initially after the surgery. Antithrombotic drugs were used in this period until weight-bearing was admitted 1 week postoperatively. The 60 mg loxoprofen sodium tablet was used three times daily until the VAS decreased to 3 on the 4th day postoperatively. The drainage tube was removed 48 h postoperatively and the compression bandage was used for 2 weeks postoperatively. The patient was discharged 2 weeks post-operation when the sutures had been removed.

The patient underwent clinical evaluation with imaging and ultrasonography at 1 month, 3 months, 6 months, and 1 year postoperatively. At 2 weeks and 1 month postoperatively, he was able to go up and down stairs without crutches. The pain (VAS: 0) had significantly relieved since the 1-month follow-up. Physical examination showed the correction of the varus deformity and the range of mobilization at the 1-month follow-up, and without changing at the last follow-up. A minor swelling in the suprapatellar and popliteal area and positive for the Ballotman's test was found at the 1-month follow-up and had disappeared since the 3 months post-operation. The X-ray showed the prosthesis's appropriate position and optimal size (Figures 2E–G). There was no loosening and subsidence of the prosthesis, no fracture around the prosthesis, and no further deterioration of the KOA. The ultrasonographic examination showed that the fluid volume decreased at the 1-month follow-up (Length: 5.6 cm, width: 1.1 cm), disappeared at the 3-month follow-up, and there was no recurrence during the following follow-up.

## Discussion

To our knowledge, there are no other reports of arthroscopic popliteal cystectomy performed concurrently with TKA or UKA. In this report, we performed concurrent arthroscopic popliteal cystectomy and UKA in a patient with a popliteal cyst in the left knee with confirmed KOA. This surgical procedure has at least 4 advantages. First, lateral osteochondral and meniscus defects will be adequately evaluated to determine whether the UKA is the best option for the patients undergoing arthroscopic popliteal cystectomy, as arthroscopy is the gold standard for diagnosing meniscal tears and osteochondral defects (14, 15). Second, meniscus trimming and forming can be performed when the lateral meniscus had a minor injury or degeneration, which cannot be treated properly when doing UKA alone. Third, an infected popliteal cyst unknown is a potential risk factor for TKA or UKA. Arthroscopic popliteal cystectomy can be performed to confirm the popliteal cyst infection with a smear or bacterial culture examination by observing the characteristics of cyst fluid or aspirating the liquid, which is difficult to identify and a contradiction for UKA (16). Finally, the procedure can relieve discomfort in the popliteal fossa region (16). The popliteal cyst symptoms may manifest as calf pain, positive Homan's sign, swelling, infection, compressive neuropathy, posterior compartment syndrome, and other findings mimicking the calf thrombophlebitis or vein thrombosis, especially if they ruptured (17–23). Therefore, cystectomy may be required, especially when the size of the popliteal cyst is greater than the baseline of 13.4 cm^2^ for the patient seeking TKA (9).

Infection is the most serious of all the potential complications during this procedure, which is also a disaster for patients receiving TKA or UKA (24, 25). The literature on whether prior arthroscopic surgery for TKA increases the risk of postoperative complications including infection is highly debated (26–29). Some surgeons recommend avoiding TKA surgery within 9 months after knee arthroscopy, and no similar study to UKA was found (29). In this study, arthroscopic cystectomy and UKA were performed concurrently. During the follow-up period, no signs of infections were found in the knee joint and popliteal region. To avoid infection, 3 points have to be paid attention to. First, use 2 g of cefazolin 30 min before and initially after the surgery. Second, another local disinfection of the surgical area with iodophor was performed between the arthroscopic cystectomy and UKA. Finally, shortening the operation time. This surgery was performed by the senior surgeon (Jin Huang), who had rich experience in arthroscopic cystectomy and UKA, and the operation time was 1 h and 40 min.

Hematoma formation is another potential complication that has to be paid attention to. In the literature, cyst wall resection has a higher rate of hematoma formation than cyst wall preservation (30). An infection may secondary to hematoma after the cystectomy. A drainage tube was placed through the inferior posteromedial portal for 48 h and a cotton pad compression dressing around the knee for 2 weeks was used to avoid this.

Stability is the third potential complication that has to be taken care of, which was one of the complications that had been highly discussed in our department before this procedure. For the arthroscopic cystectomy needs to find the entrance to the popliteal cyst by shaving the posteromedial capsular fold and enlarging the valvular area at least 5 mm (31). Though studies showed an enlargement of the capsular did not weaken the articular structure, the partial release of the medial capsule and removal of the medial meniscus during the UKA may further affect the stability in the medial compartment (13, 32). However, during the follow-up, no symptoms related to instability of the knee were found.

## Conclusion

In conclusion, this was a single case in which the patient had no recurrent cyst formation at the 1-year follow-up with adequate pain relief and no evidence of complication after the current arthroscopic cystectomy and UKA. However, certain risks require further research.

## Data Availability

The datasets presented in this study can be found in online repositories. The names of the repository/repositories and accession number(s) can be found in the article/**Supplementary material**.
