# Supplementary material for: Simultaneous arthroscopic cystectomy and unicompartmental knee arthroplasty for the management of partial knee osteoarthritis with a popliteal cyst: A case report
Source: Front Surg. 2023 Mar 30;10:1109571. doi: 10.3389/fsurg.2023.1109571 (PMC10097990; doi:10.3389/fsurg.2023.1109571)
Supplement: Supplementary file 1 [file Datasheet1.pdf]

CARE case report guidelines

CARE Checklist of information to include when writing a case report

(cc) BY-NC-ND

Topic	Item	Checklist item description	Reported on Line
Title	1	The diagnosis or intervention of primary focus followed by the words "case report"	Title, line 1-3
Key Words	2	2 to 5 key words that identify diagnoses or interventions in this case report, including "case report"	Key Words, line 31-32
Abstract (no references)	3a	Introduction: What is unique about this case and what does it add to the scientific literature?	Introduction, line 17-21
	3b	Main symptoms and/or important clinical findings	line 22-23
	3c	The main diagnoses, therapeutic interventions, and outcomes	line 23-28
	3d	Conclusion—What is the main "take-away" lesson(s) from this case?	line 29-31
Introduction	4	One or two paragraphs summarizing why this case is unique (**may include references**)	Introduction, line 35-51
Patient Information	5a	De-identified patient specific information.	line 53-54
	5b	Primary concerns and symptoms of the patient.	line 53-54
	5c	Medical, family, and psycho-social history including relevant genetic information	N/A
	5d	Relevant past interventions with outcomes	line 54
Clinical Findings	6	Describe significant physical examination (PE) and important clinical findings.	line 54-60
Timeline	7	Historical and current information from this episode of care organized as a timeline	N/A
Diagnostic Assessment	8a	Diagnostic testing (such as PE, laboratory testing, imaging, surveys).	line 54-77
	8b	Diagnostic challenges (such as access to testing, financial, or cultural)	N/A
	8c	Diagnosis (including other diagnoses considered)	line 77-78
	8d	Prognosis (such as staging in oncology) where applicable	N./A
Therapeutic Intervention	9a	Types of therapeutic intervention (such as pharmacologic, surgical, preventive, self-care)	line 79-102
	9b	Administration of therapeutic intervention (such as dosage, strength, duration)	line 98-102
	9c	Changes in therapeutic intervention (with rationale)	N/A
Follow-up and Outcomes	10a	Clinician and patient-assessed outcomes (if available)	line 103-112
	10b	Important follow-up diagnostic and other test results	line 103-112
	10c	Intervention adherence and tolerability (How was this assessed?)	N/A
	10d	Adverse and unanticipated events	N/A
Discussion	11a	A scientific discussion of the strengths AND limitations associated with this case report	line 114-124
	11b	Discussion of the relevant medical literature **with references**.	line 125-155
	11c	The scientific rationale for any conclusions (including assessment of possible causes)	N/A
	11d	The primary "take-away" lessons of this case report (without references) in a one paragraph conclusion	line 157-158
Patient Perspective	12	The patient should share their perspective in one to two paragraphs on the treatment(s) they received	N/A
Informed Consent	13	Did the patient give informed consent? Please provide if requested	Yes ☑ No ☐
