# Supplementary figures and images for: Simultaneous arthroscopic cystectomy and unicompartmental knee arthroplasty for the management of partial knee osteoarthritis with a popliteal cyst: A case report
Source: Front Surg. 2023 Mar 30;10:1109571. doi: 10.3389/fsurg.2023.1109571 (PMC10097990; doi:10.3389/fsurg.2023.1109571)

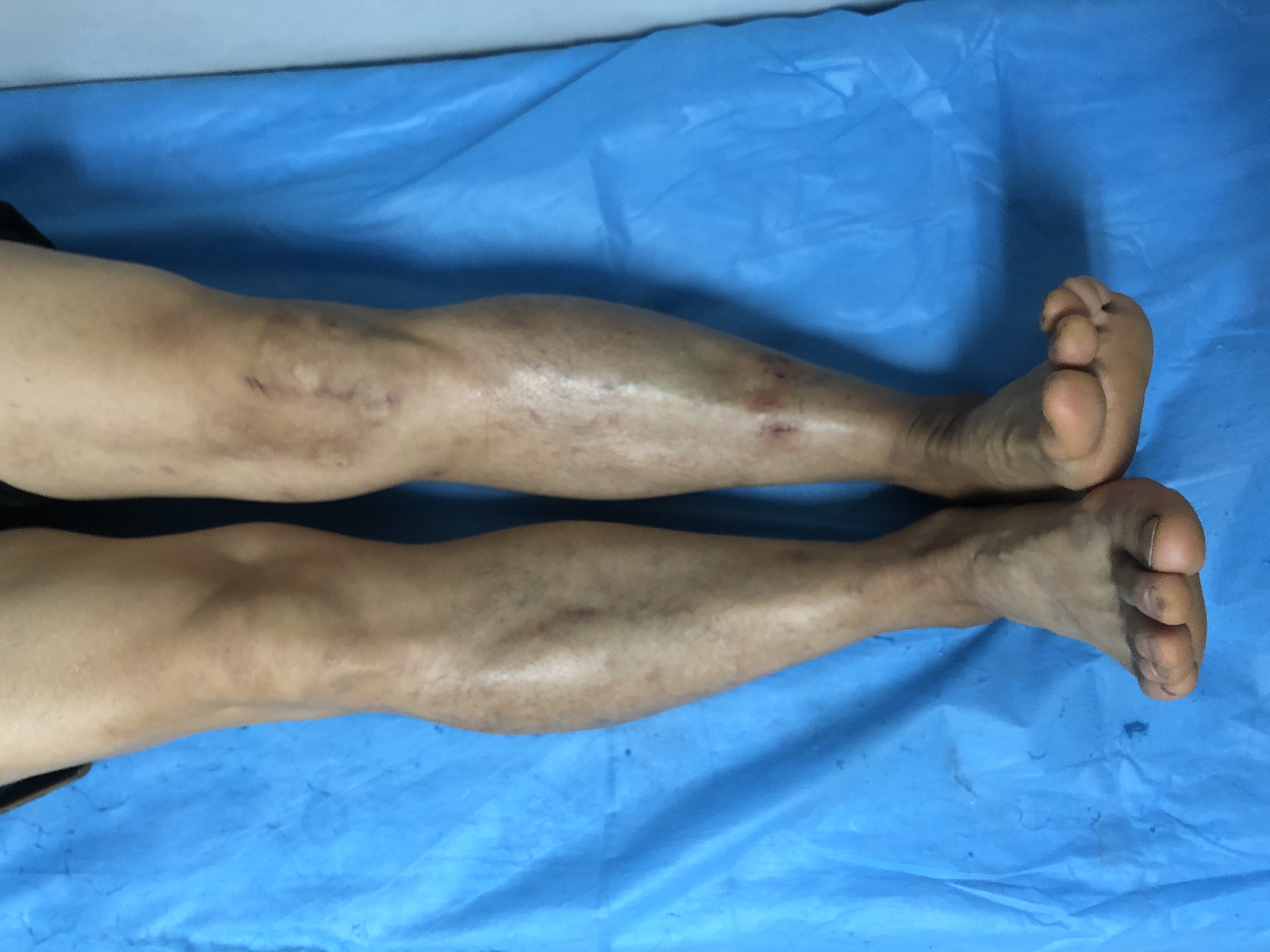

Supplement: Supplementary file 2 [file Image1.jpeg]

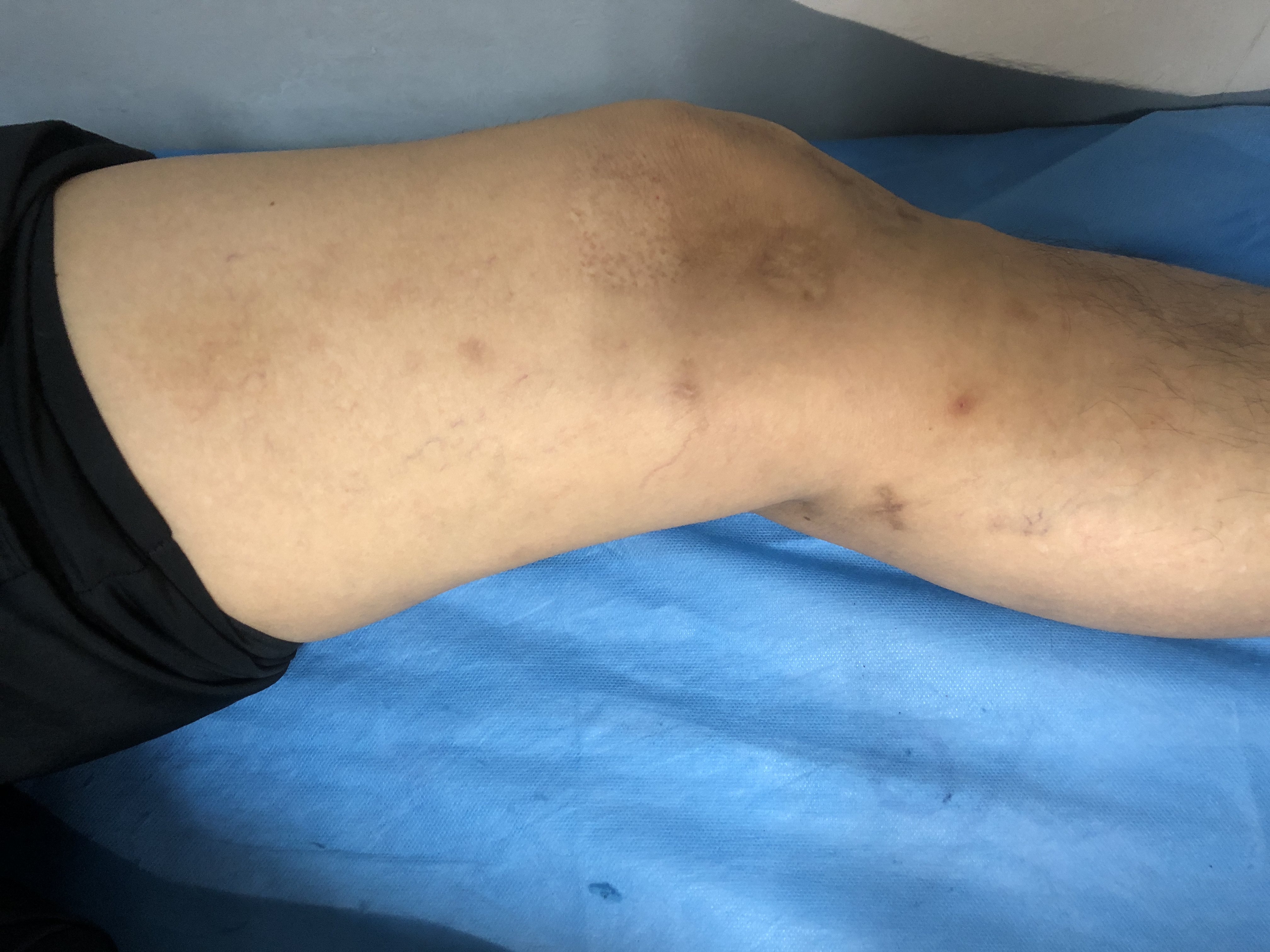

Supplement: Supplementary file 3 [file Image2.jpeg]

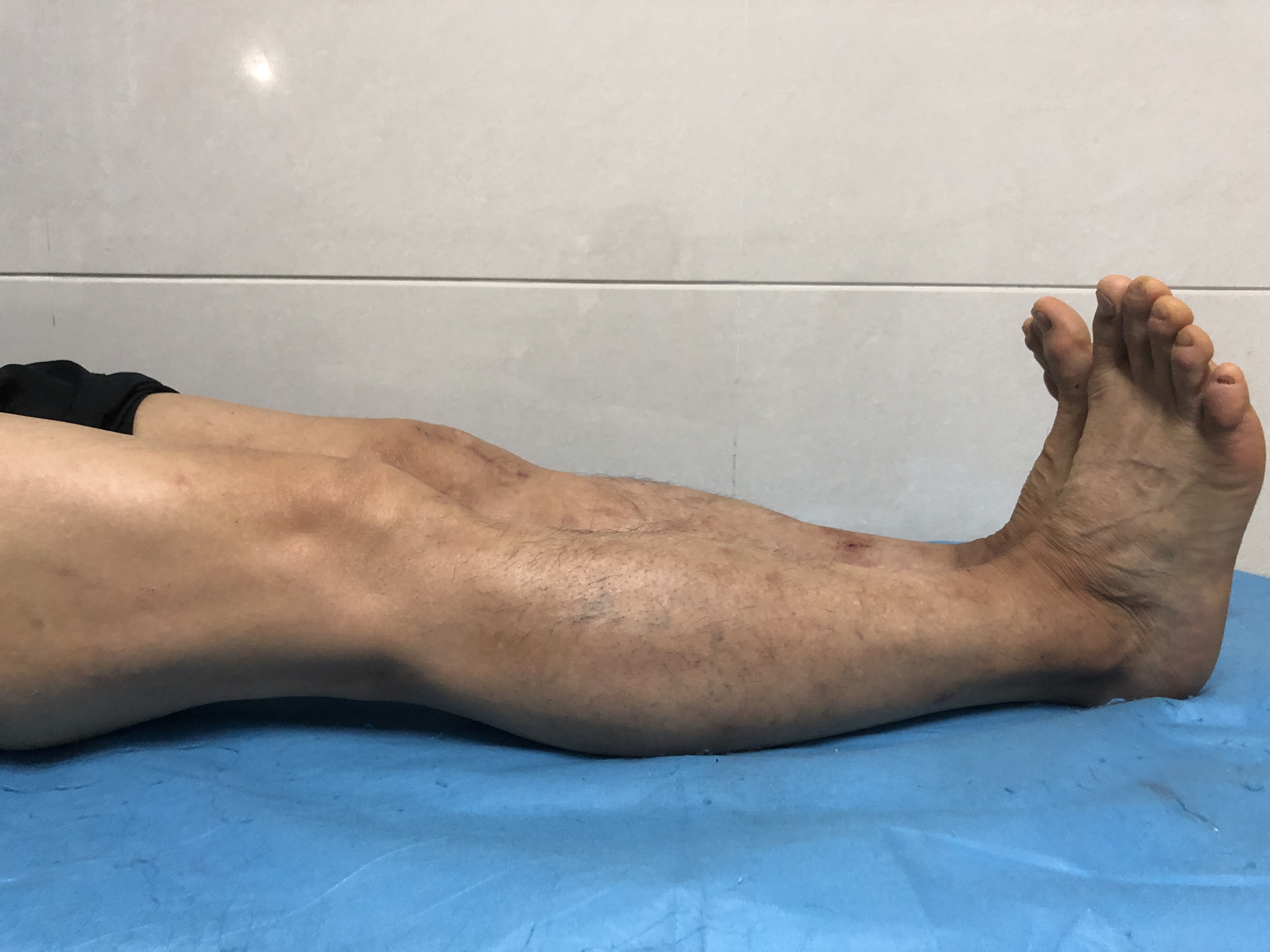

Supplement: Supplementary file 4 [file Image3.jpeg]

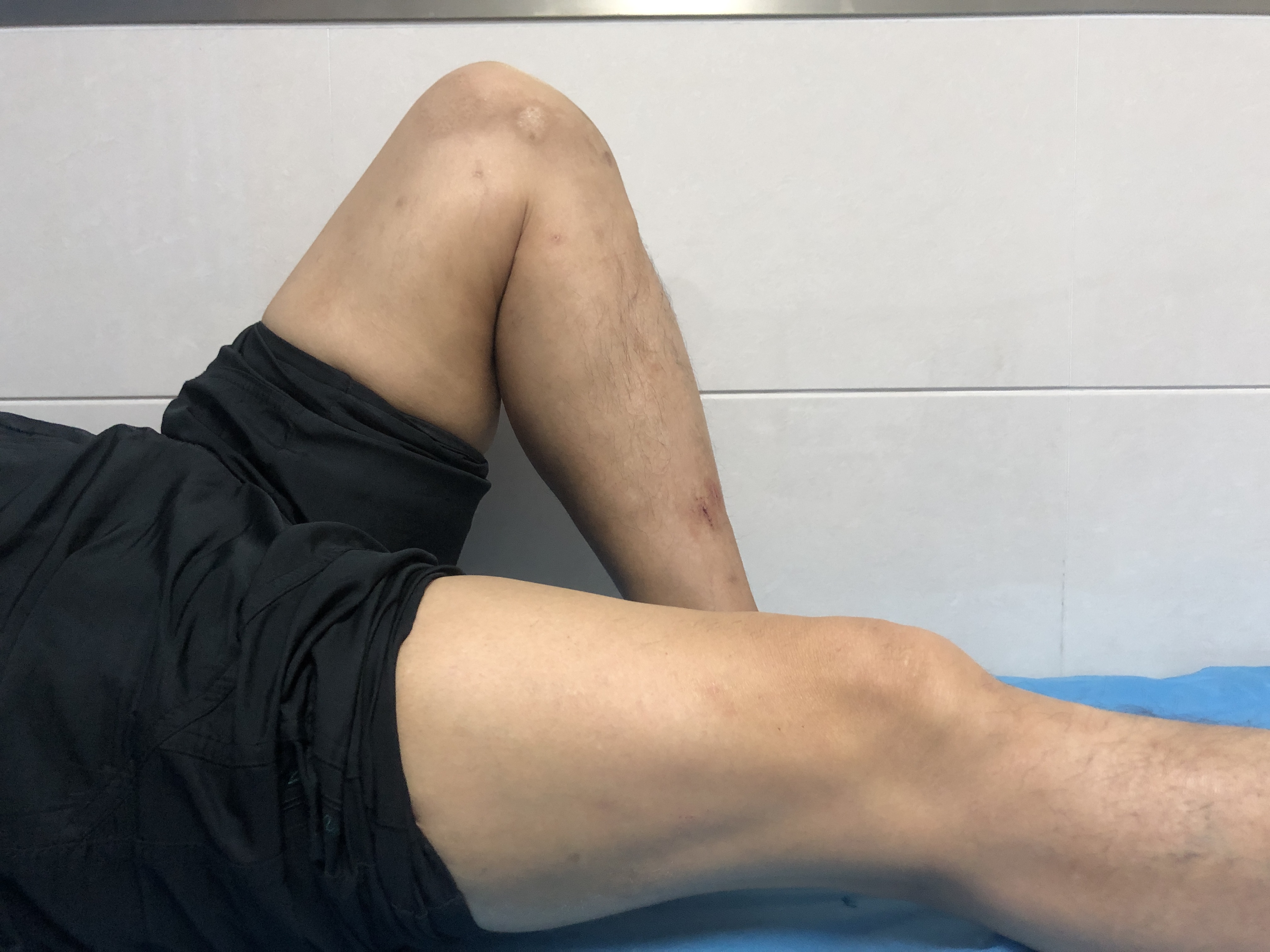

Supplement: Supplementary file 5 [file Image4.jpeg]

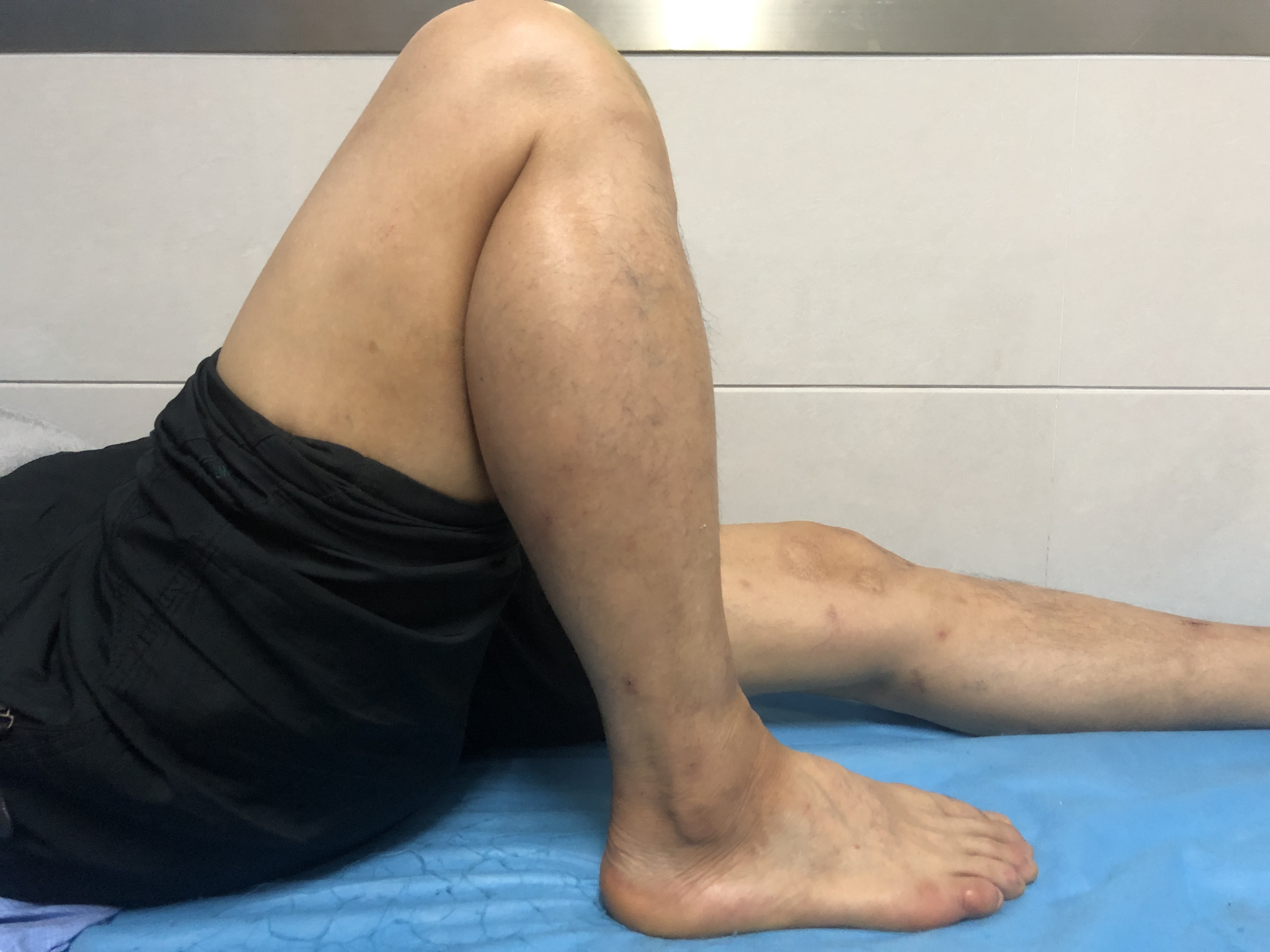

Supplement: Supplementary file 6 [file Image5.jpeg]
